# Alterations of color vision and pupillary light responses in age-related macular degeneration

**DOI:** 10.3389/fnagi.2022.933453

**Published:** 2023-01-05

**Authors:** Diego Decleva, Kallene Summer Vidal, Andre Carvalho Kreuz, Paulo Augusto Hidalgo Leite de Menezes, Dora Fix Ventura

**Affiliations:** ^1^Department of Experimental Psychology, Institute of Psychology, University of São Paulo, São Paulo, Brazil; ^2^Neuroscience and Behavior Graduate Studies Program, Institute of Psychology, University of São Paulo, São Paulo, Brazil; ^3^Prevent Senior Health Operator, São Paulo, Brazil; ^4^Service of Interdisciplinary Neuromodulation, Laboratory of Neurosciences (LIM-27), Institute of Psychiatry, Medical School, University of São Paulo, São Paulo, Brazil; ^5^Young Medical Leadership Program of National Academy of Medicine in Brazil, Rio de Janeiro, Brazil

**Keywords:** age-related macular degeneration (AMD), pupillary light reflex (PLR), intrinsically photosensitive retinal ganglion cells (ipRGC), color vision, rods (retina), cones

## Abstract

**Introduction:**

Age-related macular degeneration (AMD) is the leading cause of irreversible central vision loss in developed countries and one of the leading causes of blindness. In this work, we evaluated color vision and the pupil light reflex (PLR) to assess visual function in patients with early and neovascular AMD (NVAMD) compared with the control group.

**Methods:**

We recruited 34 early patients with dry AMD and classified them into two groups following AREDS: 13 patients with NVAMD and 24 healthy controls. Controls and patients with early dry AMD had visual acuity (VA) best or equal to 20/25 (0.098 logMAR). Color vision was assessed in controls and patients with early dry AMD using the Cambridge Color Test (CCT) 2.0 through the Trivector protocol. The PLR was evaluated using a Ganzfeld, controlled by the RETI–port system. The stimuli consisted of 1s blue (470 nm) and red (631 nm) light flashes presented alternately at 2-min intervals. To assess the cone contribution, we used a red flash at 2.4 log cd.m^–2^, with a blue background at 0.78 log cd.m^–2^. For rods, we used 470-nm flashes at –3 log cd.m^–2^, and for the melanopsin function of ipRGCs, we used 470 nm at 2.4 log cd.m^–2^.

**Results:**

Patients with early dry AMD had reduced color discrimination in all three axes: protan (*p* = 0.0087), deutan (*p* = 0.0180), and tritan (*p* = 0.0095) when compared with the control group. The PLR has also been affected in patients with early dry AMD and patients with NVAMD. The amplitude for the melanopsin-driven response was smaller in patients with early dry AMD (*p* = 0.0485) and NVAMD (*p* = 0.0035) than in the control group. The melanopsin function was lower in patients with NVAMD (*p* = 0.0290) than the control group. For the rod-driven response, the latency was lower in the NVAMD group (*p* = 0.0041) than in the control group. No changes were found in cone-driven responses between the control and AMD groups.

**Conclusion:**

Patients with early dry AMD present diffusely acquired color vision alteration detected by CCT. Rods and melanopsin contributions for PLR are affected in NVAMD. The CCT and the PLR may be considered sensitive tests to evaluate and monitor functional changes in patients with AMD.

## 1. Introduction

Age-related macular degeneration (AMD) is a progressive degenerative disease affecting the macula. Its pathogenesis involves the deposition of drusen in Bruch’s membrane and the retinal pigment epithelium (RPE), and is associated with biochemical changes that lead to the thickening of the choroidal vasculature, followed by RPE abnormality and photoreceptor dysfunction and cell death (Flores et al., 2021). AMD is the leading cause of irreversible central vision loss in developed countries and one of the leading causes of blindness worldwide, which is estimated to affect 10% of people older than 65 years and more than 25% of people older than 75 years (Ratnapriya and Chew, 2013; Wong et al., 2014; Al-Zamil and Yassin, 2017).

According to the AREDS, AMD can be classified into four stages based on the drusen deposits and the presence and/or extension of pigmentary changes (Age-Related Eye Disease Study Research Group, 2000). The first stage is normal aging, which is characterized by none or few drusen deposits smaller than 63 μm. The presence of drusen of between 63 and 124 μm with no RPE cell abnormalities is the marker for the early stage of AMD. This is the second stage, which is characterized by a slow and progressive change and loss of visual function due to choriocapillaris deterioration, atrophic loss of the outer retina, and eventually death of photoreceptor cells. The third stage is intermediate AMD, which is characterized by the presence of at least one large drusen (>124 μm) and RPE abnormalities. Early and intermediate stages are also known as dry AMD. Geographic atrophy (GA) of the fovea or any age-related characteristics of choroidal neovascularization is present in the advanced stage of AMD. The wet form of AMD is characterized by blood or serum leakage as the choroidal capillaries reach the RPE and Bruch’s membrane (BrM), causing choroidal neovascularization (NVAMD) (Downie et al., 2014; Flores et al., 2021; Yang et al., 2021; Deng et al., 2022).

The diagnostic methods for AMD include color fundus photography (CFP), fluorescein angiogenesis (FA), indocyanine green angiography (ICGA), and optical coherence tomography (OCT). These are key techniques in AMD diagnosis. CFP is commonly used to evaluate the morphology of the drusen over time; however, due to its limitations in providing details on internal retinal morphology changes, it is essential to be complemented by another method (Yang et al., 2021). The OCT is a sensitive and non-invasive method that allows cross-sectional visualization of retinal and choroidal structures. In early dry AMD, OCT can reveal the drusen size and position and also abnormalities in the RPE, which help assess the AMD evolution and its risk of progression. In NVAMD, OCT helps assess its progression by the quantification of subretinal hemorrhage and pigment epithelial detachment (Deng et al., 2022).

Although several functional changes have been reported in patients with AMD, such as dark adaptation (Chen et al., 1992; Owsley et al., 2016), spatial contrast and contrast sensitivity (Alexander et al., 1988), flicker sensitivity (Mayer et al., 1992), and photostress recovery (Brandl et al., 2022), the clinical routine examination consists of follow-up examinations aiming to evaluate changes in CFP, visual acuity (VA), and OCT (Deng et al., 2022). While these tests are useful, they do not provide enough detail about the visual system function. Thus, functional biomarkers, in addition to VA, would also be valuable as they may assist in detecting early changes. The pursuit of new sensitive methods to assess these changes may lead to better patient evaluation and early diagnosis.

The pupil light response (PLR) is a non-invasive test that assesses rod, cone, and melanopsin-driven intrinsically photosensitive retinal ganglion cells (ipRGCs) responses through flashes of chromatic light. The PLR parameters depend on the stimulus configuration – its size, brightness, duration, and wavelength as well as background illumination and retinal adaptation state (e.g., Kelbsch et al., 2019). Recently, the PLR has become a tool for aid diagnosing pathologies, physiological conditions, and, depending on the stimulus content, the cognitive or emotional status (Pinheiro and da Costa, 2021).

Previous studies have used PLR on patients with AMD to investigate ipRGC responses (Asakawa et al., 2014; Maynard et al., 2015, 2017). However, substantial differences between our study and previous studies can be noted. Previous studies have used patients classified as intermediary dry AMD (AREDS 2 and 3) and focused on the melanopsin-driven ipRGC responses using other patterns of stimuli, whereas our study was the first to use the evaluation protocol developed by Park et al. (2011) to assess rod, cone, and melanopsin-driven ipRGC responses on patients with early dry AMD (AREDS 2).

Also, in a non-invasive way, the Cambridge Color Test (CCT) assesses color discrimination thresholds using a computerized test that employs a rigorous psychophysical method and allows quantitative measures of color discrimination performance (Ventura et al., 2005). A computerized test offers the advantage of adapting and adjusting depending on the patient’s performance as well as randomizing plates to avoid repetition bias (Downie et al., 2014; Hasrod and Rubin, 2015).

Thus, these two tests can assess retinal responses in different stages and pathways, giving a wider understanding of the visual system and providing accessible tools for patient evaluation and early diagnosis, as reported in previous studies on mercury intoxication (Ventura et al., 2005; Feitosa-Santana et al., 2008), diabetes (Ventura et al., 2003; Feitosa-Santana et al., 2010), Duchenne muscular dystrophy (Costa et al., 2007), Leber’s hereditary optic neuropathy (Ventura et al., 2007), multiple sclerosis (Moura et al., 2008), autism spectrum disorders (Zachi et al., 2017), and glaucoma (Castelo-Branco et al., 2004; Duque-Chica et al., 2018).

Several studies assessed the color vision of patients with AMD using different techniques, such as arrangement tests—Farnsworth–Munsell 100 (Applegate et al., 1987), Panel D-15 (Frennesson et al., 1995), Panel D-15 e D-15d (Feigl et al., 2005), different computerized non-commercial tests computer graphics technique (Holz et al., 1995; Arden, 2004; Downie et al., 2014), and the commercially available Color Assessment and Diagnosis test (CAD) (Vemala et al., 2017). Our study is the first to use the CCT to assess color vision in patients with AMD.

In the present study, we assessed the visual function in patients with early dry and advanced neovascular AMD (NVAMD) through color vision and the PLR responses. These patient outcomes were compared with those of age-matched control subjects. We tested the hypothesis that patients with early dry AMD demonstrate dysfunction of photoreceptors, which can be assessed by sensitive methods before major changes in CFP and VA are displayed and that some changes can be also observed in patients with NVAMD.

## 2. Materials and methods

For this study, we used an observational case–control design.

### 2.1. Subjects

A total of 71 individuals—24 healthy, 34 with dry AMD, and 13 with NVAMD—were recruited at the Department of Ophthalmology in the Prevent Senior Hospital.

The subjects were divided into three groups, namely, control, early dry AMD, and NVAMD. The inclusion criteria for the control group were individuals between 50 and 85 years old with best-corrected VA equal to or better than 20/30 (0.17 logMAR) in the studied eye. The inclusion criteria for the early dry AMD group were patients with a diagnosis of AMD classified as AREDS 2 by an ophthalmologist and with best-corrected visual acuity equal to or better than 20/30 (0.17 logMAR) in the studied eye. The inclusion criteria for NVAMD group were patients with a diagnosis of NVAMD with active neovascularization and intra-vitreous injection indication. All subjects underwent a complete ophthalmologic evaluation, including a medical history review, best-corrected visual acuity measurement, biomicroscopy, refraction, and intraocular pressure (IOP) measurement. Subjects were included after confirmation of the diagnosis by spectral-domain OCT (Cirrus HD OCT, Model 4000; Carl Zeiss Meditec, Dublin, CA, USA).

The exclusion criteria were a concomitant ocular disease, a history of retina and/or glaucoma surgery, altered IOP or abnormal excavation of the optic nerve, neurological or psychiatric illness, diabetes, smoking or alcohol abuse, or the use of any medication that could potentially affect the pupillary response.

Only one eye of each patient was evaluated. The eye was chosen by the best VA, and in case of the same VA in both eyes, the eye was randomly selected. In the case where both eyes had dry AMD, the least affected eye was chosen. In the case of detection of early dry AMD in one eye and NVAMD in the other eye, the chosen eye was the eye with NVAMD. For NVAMD, all tests were performed prior to any anti-VEGF injection.

Informed consent was obtained from all subjects before participation. The protocol was approved by the ethics committees of the Psychology Institute of the University of Sao Paulo (2.065.527) and the Prevent Senior (4.01.N.2016.12.01). All procedures adhered to the tenets of the Declaration of Helsinki.

### 2.2. Color vision

Color discrimination thresholds were measured using the CCT v2.0 with stimuli generated by a ViSaGe 2/5 graphics card (Cambridge Research Systems, Rochester, England, UK) and a gamma-corrected Sony FD Trinitron color monitor (Model G420 Trinitron, Multiscan; Sony Corporation, Tokyo, Japan).

The CCT is based on the same principle as the Ishihara pseudoisochromatic plates. It uses spatial noise and luminance noise in the stimuli to eliminate the influence of spatial contour or luminance clues in color discrimination. A mosaic of small circles of several sizes and luminance composes the entire image presented to the subject. A subset of these circles, which forms a Landolt “C,” is shown at a different chromaticity from the rest of the image. The Landolt “C” subset constitutes the target, remaining area, and background. The subjects were tested in a dark room, seated at 3 m from the monitor.

Monocular tests were performed only in the chosen eye. The target was randomly presented with the gap of the “C” at one of the following positions: up, down, right, or left in a 4 alternative forced choice. The subjects were instructed to identify the position of the “C” gap and report it verbally to the experimenter, who entered the patient response in a remote response box control (CT6, CRS). This was a precaution to avoid misleading results by mistakes in the use of the response box. Only subjects in the control and early dry AMD groups were tested in the CCT since the low vision in NVAMD could affect the patient performance.

Color vision was assessed through the Trivector protocol, which enables rapid measurement of color discrimination thresholds along the protan, deutan, and tritan confusion axes. Thresholds may be measured in less than 10 min. Thresholds are expressed in vector length units (u’v’*10^–4^) of the CIE 1976 chromaticity diagram.

The CCT has been widely used for a wide range of health conditions; still, its limitation remains the same as for most color tests as it also relies on the VA for reliable results. Although some color vision tests are available for patients with lower vision, only few tests are suitable for patients with severely impaired visual acuity or visual field (Jolly et al., 2021). Thus, we focused on early signs of color vision alterations in patients in the first stage of the disease.

### 2.3. Pupillary light reflex (PLR)

#### 2.3.1. Procedure

The PLR was evaluated using a Ganzfeld controlled by an RETI–port pupillometer (Roland Consult, Germany). The stimuli consisted of 1s blue (470 nm) and red (631 nm) light flashes presented alternately at 2-min intervals. To assess the contribution of cone photoreceptors, we used a 631-nm flash at 2.4 log cd.m^–2^, with a blue background (470 nm at 0.78 log cd.m^–2^). For rods, we used 470-nm flashes at –3 log cd.m^–2^ without background light. For the melanopsin response measured by the post-illumination pupil response (PIPR), we used 470-nm flashes at 2.4 log cd.m^–2^, also with no background light. A photopically matched stimulus at 631 nm, a region that causes no or minimal melanopsin activation, was also presented for comparison. The difference between the response to the red and blue lights evidences the melanopsin response in the PLR. This protocol was based on Park et al. (2011).

Measurements of the PLR were carried out with an infrared eye-tracking camera system at a 60-Hz sample rate of real-time pupil recording. The subjects were dark-adapted for 10 min prior to the beginning of the measurements.

#### 2.3.2. PLR analysis

The PLR data were analyzed offline using a self-written script programed in OriginPro^^®^^ (v. 8.5.1 SR2, OriginLab Corp., Northampton, MA, USA). First, a median filter with a 300-ms time window was applied to remove eye blinks. In the case of long eye blinks or eye closure, the artifact was removed manually.

**FIGURE 1:**
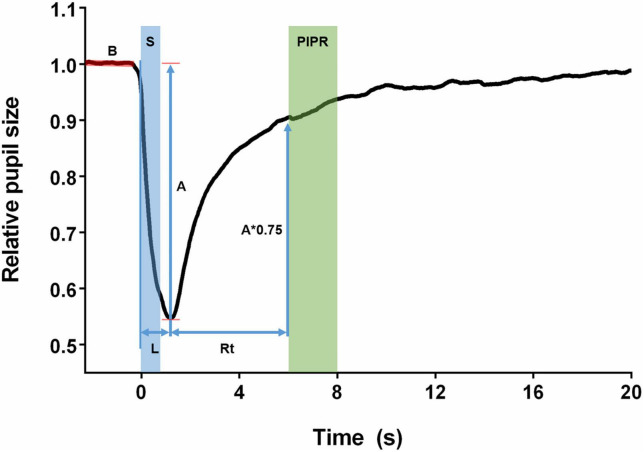
Features of the pupillary light reflex. The baseline (B) was obtained as the median of the pupil size of the 3 s before the stimuli. The stimulus (S) was presented as a step of light with 1-s duration. Amplitude (A) was defined as the maximum constriction reached by the pupil after the stimulus. Latency (L) was the time between the beginning of the stimulus and the moment when the pupil reached its maximum constriction. Recovery time was calculated from the pupil maximum constriction until the pupil had returned to 75% of its baseline size (A*0.75). The post-illumination pupil response (PIPR) was calculated as the median pupil size recorded in the period of 6–8 s after the start of the stimulus.

The parameters obtained are shown in Figure 1. The baseline was obtained by the median pupil size of filtered PLRs of the 3 s prior to each stimulus onset. The PLRs were then normalized by the baseline pupil size. The relative pupil size was defined as the absolute pupil size (mm)/baseline pupil size (mm). The pupil size amplitude was obtained as the baseline divided by the minimum pupil size. Peak latency was the time for the pupil to reach its minimum size. Recovery time was the time for the pupil to reach 75% of its baseline size. The PIPR was expressed as the median pupil size from 6 to 8 s after the stimulus offset. The PIPR parameter was determined only for the melanopsin-activating stimulus (high-intensity blue light) as this parameter reflects the melanopsin function of the ipRGC, while the other parameters were measured for all stimuli (Kelbsch et al., 2019).

#### 2.3.3. Optical coherence tomography

The subjects underwent spectral-domain OCT (SD-OCT) scanning using a commercially available device (Cirrus HD-OCT, Zeiss, Germany). The scanning protocol involved the acquisition of a 6 × 6-mm cube scan of the macula at a scan density of 512 × 128 pixels. The criteria for acceptable OCT images are as follows: the absence of large eye movements, defined as an abrupt shift completely disconnecting a large retinal vessel; consistent signal intensity level across the scan; and absence of the black band.

### 2.4. Statistics

The data obtained were analyzed to identify and remove outliers through the robust regression and outlier removal (ROUT) test, using *Q* = 0.5%. After the ROUT test, the data were tested for normality using the Shapiro–Wilk test. The pupillometric data were analyzed using ANOVA with Dunnett’s test for multiple comparisons when it passed the normality test or Dunn’s test when the data had not passed the normality test. As the CCT was performed only in the control and early dry AMD groups, the data were compared using the *t*-test. A *p*-value of <0.05 (two-tailed) was considered statistically significant.

## 3. Results

In this work, 119 subjects were evaluated; however, 71 subjects were included in the final sample study, with 24 subjects in the control group, 34 subjects in the early dry AMD group, and 13 subjects in the NVAMD group (Table 1). We excluded 48 subjects due to previous eye conditions such as glaucoma, eye surgery, and diabetic retinopathy and also due to the use of medication that could potentially affect the pupillary response.

**TABLE 1:** Demographic data for control, early, and NVAMD groups.

	Control	Early dry AMD	NVAMD	*P*-value
*N*	24	34	13	
Age-years (SD)	73.3 (6.9)	72.5 (7.7)	80.2 (5.2)	0.0064
Gender (%)	F 52.63	F 73.33	F 66.66	0.3354
	M 47.36	M 26.66	M 33.33	
AV (LogMAR)	0.055	0.098	1.004	0.0002
**Macular OCT**				
MT (μ m)	249.33 (35.30)	252.77 (33.08)	302.33 (66.09)	0.0142
MCV (mm^2^)	10.22 (0.39)	10.08 (0.60)	10.73 (0.74)	0.4841
**Pupil baseline size**				
Rod-driven (mm)	4.58 (0.83)	4.43 (0.76)	4.31 (0.71)	0.5422
Cone-driven (mm)	2.62 (0.44)	2.84 (0.51)	2.77 (0.54)	0.2874
Melanopsin-driven (mm)	4.68 (0.84)	4.68 (0.80)	4.40 (0.81)	0.4544

NVAMD, neovascular age-related macular degeneration; F, female; M, male; MT, macular thickness; MCV, macular cube volume; VA, visual acuity; AMD, age-related macular degeneration; ipRGCs, intrinsically photosensitive retinal ganglion cells.

### 3.1. Color vision

**FIGURE 2:**
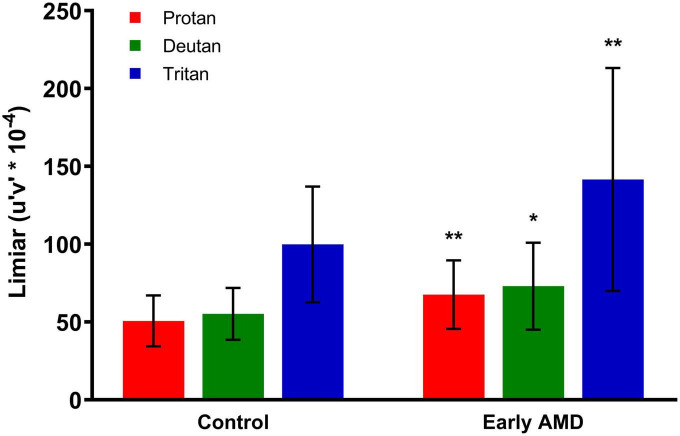
Cambridge Color Test (CCT) results. The performance for color discrimination is represented by color discrimination thresholds along protan, deutan, and tritan axes for the control and early dry age-related macular degeneration (AMD) groups. Statistically significant comparisons between the respective color confusion axes in the two groups are marked with asterisks. Unpaired *t*-test. **Protan (*p* = 0.0087), *deutan (*p* = 0.0180), **tritan (*p* = 0.0095). CCT, Cambridge Color Test.

When compared with the control group, patients with early dry AMD had worse performance in color discrimination, expressed by longer vector length between the threshold and the background chromaticities, in all three axes, as shown in Figure 2.

The threshold in the protan axis in the control group was 50.76 ± 16.35 compared with 67.66 ± 22.02 for the early dry AMD group (*p* = 0.0087). The deutan axis threshold in the control group was 55.22 ± 16.66 compared with 73.07 ± 27.95 for the early dry AMD group (*p* = 0.0180). The tritan axis threshold in the control group was 99.88 ± 37.20 compared with 152.8 ± 92.52 in the early dry AMD group (*p* = 0.0095).

### 3.2. PLR

**FIGURE 3:**
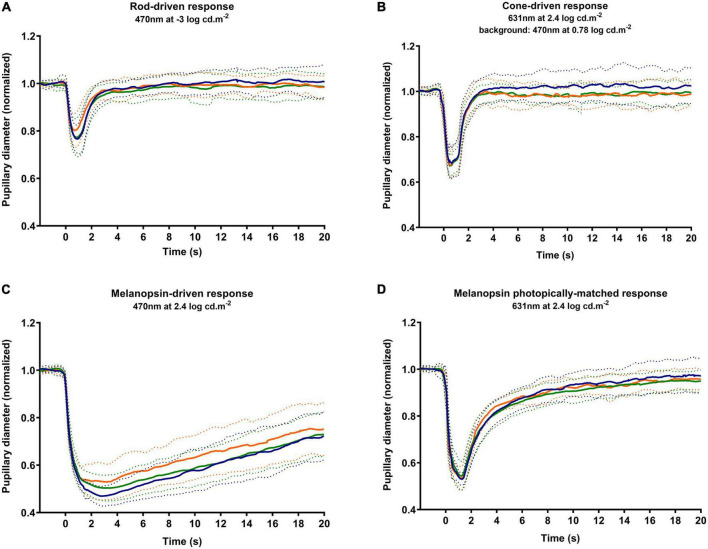
Average PLRs for each group. The average response is marked as a solid line, and the standard deviation is marked as dotted lines. The responses obtained by the tested stimuli in control (blue markers), early dry age-related macular degeneration (AMD) (green markers), and NVAMD (orange markers) groups were the rod-driven response **(A)**, cone-driven response **(B)**, melanopsin-driven response **(C)**, and a melanopsin photopically matched stimulus **(D)**. PLR, pupil light reflex; NVAMD, neovascular age-related macular degeneration.

**FIGURE 4:**
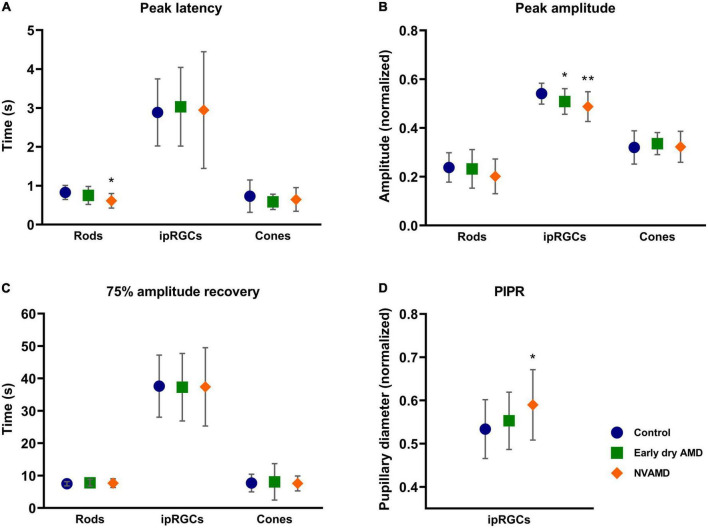
Average and standard deviation of peak latency **(A)**, amplitude **(B)**, 75% amplitude recovery time **(C)**, and post-illumination pupil response (PIPR) **(D)** for early dry age-related macular degeneration (AMD) (green square), NVAMD (orange diamond), and the control groups (blue circle) for rod-, melanopsin-, and cone-driven stimuli. NVAMD, neovascular age-related macular degeneration. *p < 0.05, **p < 0.01.

Figure 3 shows the average response to rod-driven (Figure 3A), cone-driven (Figure 3B), melanopsin-driven (Figure 3C), and melanopsin photopically matched stimulus (Figure 3D) conditions. The analysis of parameters as the baseline, amplitude, latency, PIPR, and recovery time is presented in Figure 4 and described.

Before every stimulus, the baseline pupil size was measured. No differences were observed among the groups. This measurement aimed at ensuring that all groups had reached the same dark adaptation level since the testing protocol and eventual autonomic changes might alter pupil size and its dark adaptation time.

#### 3.2.1. Rod-driven condition

We observed that the NVAMD group had a significantly shorter latency (*p* = 0.0041) than the control group (Figure 4A). However, no differences between the early dry AMD and control groups were found (*p* = 0.2992).

The constriction amplitude for rod-driven response (Figure 4B) showed no differences between early dry AMD (*p* = 0.9384) and NVAMD (*p* = 0.2093) groups when compared with the control group. Likewise, the recovery time also showed no difference between early dry AMD (*p* = 0.7829) and NVAMD (*p* ≥ 0.9999) groups when compared with the control group (Figure 4C).

#### 3.2.2. Cone-driven condition

No differences were found for cone-driven response parameters. The peak latency showed no difference between early dry AMD (*p* = 0.3745) and NVAMD (*p* = 0.9795) groups when compared with the control group (Figure 4A). Response amplitude also did not differ statistically between early dry AMD (*p* = 0.5259), NVAMD (*p* = 0.9886), and control groups (Figure 4B). No difference in the recovery time was observed between early dry AMD (*p* = 0.7854), NVAMD (*p* ≥ 0.9999), and control groups for the cone-driven response (Figure 4C).

#### 3.2.3. Melanopsin-driven condition

For the melanopsin-driven response condition, we observed statistical differences in the response amplitude between both early dry AMD (*p* = 0.0485) and NVAMD (*p* = 0.0035) groups relative to the control group (Figure 4B). For the PIPR, a difference was observed between NVAMD (*p* = 0.0290) and the control groups, but not for the early dry AMD group (*p* = 0.4595; Figure 4D).

No difference was found for latency between early dry AMD (*p* = 0.7739), NVAMD (*p* => 0.9999), and control groups (Figure 4A). Also, the time to recover for the early dry AMD (*p* = 0.9916) and NVAMD (*p* = 0.9973) groups did not differ from that of the control group (Figure 4C).

The melanopsin-matched condition showed no difference in the peak latency between early dry AMD (*p* = 0.9087), NVAMD (*p* = 0.6621), and control groups. The peak response amplitude also did not differ statistically between early dry AMD (*p* = 0.3915), NVAMD (*p* = 0.5158), and control groups. Similarly, the recovery time also presented no difference between early dry AMD (*p* = 0.2963) and NVAMD (*p* = 0.9039) when compared with the control group.

## 4. Discussion

The present study investigated color vision alterations using the CCT and PLR in early dry AMD and NVAMD compared with a control group. A diffuse color vision discrimination loss in patients with early dry AMD was found with the CCT, despite the patients’ good VA. In the PLR, the rod and melanopsin response contributions were affected in the NVAMD group.

The CCT results agree with the majority of studies that showed color vision alterations in early dry AMD detected by other tests (Applegate et al., 1987; Frennesson et al., 1995; Holz et al., 1995; Arden, 2004; Feigl et al., 2005; Downie et al., 2014; Vemala et al., 2017). One study using the CAD test showed alterations in red/green and yellow/blue in dry AMD when compared with the control group, mainly in patients with reticular pseudodrusen observed in the OCT (Vemala et al., 2017). A study using the Farnsworth–Munsell 100 arrangement test did not show any color vision alterations in dry AMD compared with the control group (Midena et al., 1997), while in another study, tritan alterations in dry AMD were reported using the Panel D-15 test (Feigl et al., 2005). In intermediate AMD stages, in addition to the color vision impairment, a luminance alteration was observed (Downie et al., 2014), which indicates that color vision alterations may be accompanied by or precede other visual function disorders (Holz et al., 1995).

Some inconsistencies in the literature may have been due to the use of different techniques to assess the color vision function in patients with AMD. The CCT has been considered a reliable and sensitive test to evaluate color vision in different diseases (Ventura et al., 2003, 2005, 2007; Castelo-Branco et al., 2004; Feitosa-Santana et al., 2008, 2010; Moura et al., 2008; Zachi et al., 2017; Duque-Chica et al., 2018). The results of the present study indicate that the CCT could be a functional test to evaluate AMD progression before the VA decline.

The PLR has been used to study several ophthalmological and non-ophthalmological diseases (Bremner, 2009; Roecklein et al., 2013; Laurenzo et al., 2016; Park et al., 2016; Hall and Chilcott, 2018; Chougule et al., 2019). Previous studies with PLR in AMD showed alterations in several parameters such as latency, amplitude, time for maximum constriction, maximum constriction velocity, and baseline pupil radius when compared with a control group; however, these studies included more advanced NVAMD (Brozou et al., 2009; Sabeti et al., 2011, 2013; Rosli et al., 2012; Asakawa et al., 2014).

In the present data, we described photoreceptor dysfunctions in both early dry AMD and NVAMD. Our study showed that rod contributions to the PLR are affected in NVAMD, while cone contributions did not differ between the groups. Functional evaluations in patients with AMD had shown that impairment in rod responses may be more extensive than cone dysfunction in AMD (Chen et al., 1992; Scholl et al., 2004). Also, this finding is in agreement with histologic data that have demonstrated a preferential loss of rods in AMD (Curcio et al., 1993, 1996). This might partially explain why PLR detected alterations in the rod but not in cone responses in our study. However, color vision loss detected by the CCT showed cone dysfunctions in patients with early dry AMD. Our hypothesis is that PLR is more sensitive in detecting rod than cone abnormalities, and the CCT could be a better test to evaluate cone responses.

The PLR results also showed abnormalities in the ipRGC contributions to the NVAMD group. In the early dry AMD, for the melanopsin-driven response, we observed a smaller amplitude than the control group. Although photoreceptors also partake in the transient phase of the pupillary constriction, and therefore their contribution must also be taken into account, when presented with a photopically matched stimulus, no differences in both AMD groups have been observed when compared with a control group.

Using the same stimulus condition, we observed a smaller transient peak amplitude and a smaller PIPR, suggesting alterations in the ipRGC function. This can also be corroborated by the photopically matched stimulus used to isolate the melanopsin response. Maynard et al. (2015) showed that PIPR and amplitude are affected in the early dry AMD group. However, it is valid to note that the reduced PIPR in the NVAMD group may reflect the overall reduction of their PLR, rather than a reduced melanopsin response.

Different studies showed changes in the melanopsin function of the ipRGCs not only in inner retina disorders such as glaucoma (Kelbsch et al., 2016) and ischemic optic neuropathy (Herbst et al., 2013) but also in outer retina diseases such as diabetic retinopathy (Obara et al., 2017), and early and late AMD (Maynard et al., 2015, 2017). Likewise, changes in the melanopsin function were reported in neurological and psychiatric conditions including Parkinson’s disease (Joyce et al., 2018), multiple sclerosis (Meltzer et al., 2017), and depressive disorder (Feigl et al., 2018). Maynard et al. (2015) argued that ipRGCs are more affected in central retinal disorders since ipRGCs have the highest density in the paracentral retina, different from that observed in optic nerve degenerations where ipRGCs are more resistant than other retinal ganglion cells (Kawasaki et al., 2010; La Morgia et al., 2010; Moura et al., 2013). However, the mechanism that involves ipRGC impairment in AMD group is unclear. More studies are needed for its elucidation.

A possible limitation of the present study is the age difference between the control and NVAMD groups (Table 1). As reported, the pupil size decreases significantly with age, and such changes must be controlled in aging studies on visual functions (Telek, 2018). However, a study showed that there is no effect of age on ipRGC inputs in the human pupil control pathway (Adhikari et al., 2015); also, in our study, the baseline pupil size for each photoreceptor response did not present differences among the groups (Table 1), so we believe that the difference in age among groups was not a bias.

Also, in this study, we used a higher luminance to obtain a cone-driven response than in some previous works (2.4 log cd.m^–2^ vs. 1 log cd.m^–2^) (Park et al., 2017; Joyce et al., 2018; La Morgia et al., 2022). However, this luminance is still slightly lower than followed in a protocol previously established by Park et al. (2011), who used 2.6 log cd.m^–2^. Increasing the luminance ensures minimal rod response influence on the cone pathway. Moreover, Park et al. (2011) showed that cones present a better response at higher intensities, and as cones do not saturate as intensity is increased (Shevell, 1977), we believe that the difference did not have a significant impact on the outcome.

On the other hand, one of the strengths is that in our study, we selected patients in the earliest stage of AMD (AREDS 2) meaning that CCT and PLR might become useful tools to evaluate AMD progression from early to late stages. Also, this is the first report to use the CCT to evaluate acquired color vision alterations in patients with AMD. Our results show that both tests are feasible and could be used as biomarkers of AMD progression. Finally, the CCT was not performed in patients with NVAMD due to their lower visual acuity. Although the low-vision Cambridge Color Test (lvCCT) can be used as an alternative in these cases (Simunovic et al., 1998; Jolly et al., 2022), our focus was to evaluate early changes before any significant visual alteration.

## 5. Conclusion

Our results showed that patients with early dry AMD present diffusely acquired color vision alteration detected by the CCT. Rod and ipRGC contributions to the PLR are affected in early dry AMD and NVAMD groups. The CCT and PLR may be considered useful tests to evaluate and monitor functional changes in AMD.

## Data availability statement

The raw data supporting the conclusions of this article will be made available by the authors, without undue reservation.

## Ethics statement

The studies involving human participants were reviewed and approved by Psychology Institute of the University of São Paulo Ethical Committee Board and the Prevent Senior Ethical Committee Board. The patients/participants provided their written informed consent to participate in this study.

## Author contributions

DD, KV, and DV conceived the study and analyzed the data. DV obtained funding and reviewed the manuscript. DD and KV performed the research and wrote the manuscript. AK performed OCT analysis. PM performed patient recruitment and triage. All authors approved the submitted version.
